# MOBIDB in 2025: integrating ensemble properties and function annotations for intrinsically disordered proteins

**DOI:** 10.1093/nar/gkae969

**Published:** 2024-10-29

**Authors:** Damiano Piovesan, Alessio Del Conte, Mahta Mehdiabadi, Maria Cristina Aspromonte, Matthias Blum, Giulio Tesei, Sören von Bülow, Kresten Lindorff-Larsen, Silvio C E Tosatto

**Affiliations:** Department of Biomedical Sciences, University of Padova, Padua 35131, Italy; Department of Biomedical Sciences, University of Padova, Padua 35131, Italy; Department of Biomedical Sciences, University of Padova, Padua 35131, Italy; Department of Biomedical Sciences, University of Padova, Padua 35131, Italy; European Molecular Biology Laboratory, European Bioinformatics Institute (EMBL-EBI), Wellcome Genome Campus, Hinxton, Cambridgeshire CB10 1SD, UK; Structural Biology and NMR Laboratory, Linderstrøm-Lang Centre for Protein Science, Department of Biology, University of Copenhagen, Copenhagen, Denmark; Structural Biology and NMR Laboratory, Linderstrøm-Lang Centre for Protein Science, Department of Biology, University of Copenhagen, Copenhagen, Denmark; Structural Biology and NMR Laboratory, Linderstrøm-Lang Centre for Protein Science, Department of Biology, University of Copenhagen, Copenhagen, Denmark; Department of Biomedical Sciences, University of Padova, Padua 35131, Italy; Institute of Biomembranes, Bioenergetics and Molecular Biotechnologies, National Research Council (CNR-IBIOM), Bari, Italy

## Abstract

The MobiDB database (URL: https://mobidb.org/) aims to provide structural and functional information about intrinsic protein disorder, aggregating annotations from the literature, experimental data, and predictions for all known protein sequences. Here, we describe the improvements made to our resource to capture more information, simplify access to the aggregated data, and increase documentation of all MobiDB features. Compared to the previous release, all underlying pipeline modules were updated. The prediction module is ten times faster and can detect if a predicted disordered region is structurally extended or compact. The PDB component is now able to process large cryo-EM structures extending the number of processed entries. The entry page has been restyled to highlight functional aspects of disorder and all graphical modules have been completely reimplemented for better flexibility and faster rendering. The server has been improved to optimise bulk downloads. Annotation provenance has been standardised by adopting ECO terms. Finally, we propagated disorder function (IDPO and GO terms) from the DisProt database exploiting sequence similarity and protein embeddings. These improvements, along with the addition of comprehensive training material, offer a more intuitive interface and novel functional knowledge about intrinsic disorder.

## Graphical Abstract

**Graphical Abstract:**
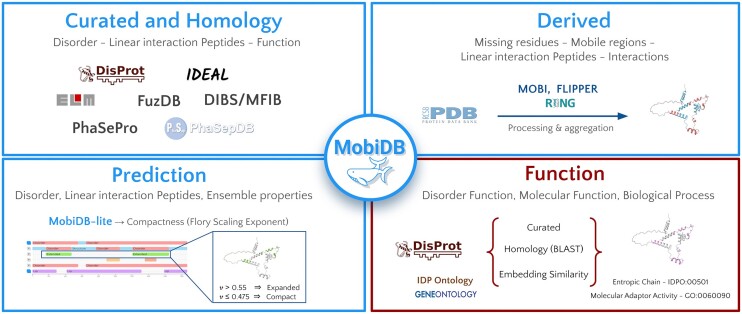

## Introduction

About 30% of eukaryotic proteomes are composed of intrinsically disordered (ID) proteins or regions, forming dynamic conformational ensembles that challenge the traditional sequence-structure-function paradigm (1). These proteins play crucial roles in cellular processes and their dysfunction is linked to neurodegenerative diseases and cancers (2).

ID proteins and regions exhibit a diverse array of rapidly interconverting structures in solution and often play important roles in the formation and dynamics of biomolecular condensates within cells (3). Despite lacking stable folded states, ID regions possess local and global order, influencing their dimensions, shape, interactions with other proteins, and biological functions.

ID research delves into various experimental domains dedicated to exploring protein structure and function, spanning disciplines such as structural biology, biophysics, biochemistry, cell biology, proteomics, among the others. Traditional methods like X-Ray crystallography, while fundamental for structural characterization, fall short in capturing the dynamic nature of ID regions. To comprehend the structural nuances of ID regions, researchers employ an array of biophysical techniques such as nuclear magnetic resonance (NMR), small-angle X-ray scattering (SAXS), circular dichroism (CD) and Förster resonance energy transfer (FRET). By harmonising insights from these diverse methodologies, a comprehensive model of ID regions structure and dynamics emerges.

Each of these experimental techniques necessitates manual interpretation because varying experimental conditions can result in different structural states. Although dedicated databases gather primary data and pertinent metadata from specific sources, they struggle to integrate their content uniformly (4,5).

MobiDB is a unique resource that serves as a comprehensive knowledgebase of ID proteins and regions (6) that was first published in 2012 (7) with the aim of filling this gap. MobiDB strives to generate new knowledge and capture the functional significance of disordered regions by processing and combining complementary sources of information. It aggregates disorder annotations from the literature and experimental evidence, along with predictions, for all known protein sequences. Curated data from literature evidence is directly pulled from third-party resources, building on extensive and fruitful collaborations with many databases such as DisProt (8), PED (9), IDEAL (10), ELM (11), FuzDB (12) and more.

MobiDB utilises UniProtKB sequences and identifiers (13), and its disorder predictions are propagated to other core data resources through the MobiDB-lite software (14,15). MobiDB is a member of the InterPro consortium (16) and MobiDB-lite is also integrated in the InterProScan software (17). UniProtKB (13) uses MobiDB-lite predictions to define rules for the automatic annotation of various sequence patterns. PDBe and PDBe-KB (18,19) provide MobiDB annotations for ‘*flexibility*’ in protein structures. All annotations, including derived annotations from the PDB and AlphaFoldDB (20) as well as manually curated evidence imported from member databases are exposed via an API in a standardised way.

MobiDB celebrated ten years in the previous publication (6) where a historical perspective is provided. In this work, we describe the improvements made to our resource to capture more information, simplify access to the aggregated data, and increase documentation for an informed usage of all MobiDB features.

Compared to the previous version we updated all the underlying pipeline modules in order to process more data, to be faster, and to include additional functional features exploiting new methods for the prediction of ensemble properties. The latter is the result of the integration of a compactness predictor, developed by Tesei *et al.* (21), into our pipeline. This machine learning model was trained on data from molecular simulations of human ID regions and predicts the apparent Flory scaling exponent, based on sequence only.

## Progress and new features

The MobiDB data generation pipeline is composed of two distinct components, i) an integration pipeline which is responsible for retrieving, process, and aggregating information from other resources including manually curated data from member databases and PDB data; and ii) the MobiDB-lite software which generates all disorder predictions.

The MobiDB-lite software (15) is developed in collaboration with the InterPro (16) team, it is Open Source and available at URL: https://github.com/BioComputingUP/MobiDB-lite.

The integration pipeline includes the RING (22), MOBI (23) and FLIPPER (24) software for the processing of PDB structures and the calculation of non-covalent interactions, mobile regions in NMR ensembles and linear interacting peptides (LIPs) in protein complexes, respectively. Moreover, we integrated a novel module that provides Gene Ontology (GO) (25) and Intrinsically Disordered Proteins Ontology (IDPO) terms for ID regions as described in the function annotation paragraph below.

### Derived data

The MobiDB integration pipeline has been updated in order to maximise the coverage of processed PDB entries. The information extracted from PDB entries which is translated into ‘*derived*’ data in MobiDB has been improved by implementing the processing of mmCIF files and removing all dependencies from third-party software. The PDB mmCIF format, among other advantages, separates the identifiers of the ligands from polymeric chains, therefore simplifying the identification of context-dependent inter-chain interactions. Also, mmCIF files are not limited in the number of structure chains therefore increasing the range of processable entries which now include large macro-molecular complexes as provided by cryo-EM experiments. The number of processed proteins from PDB data is 65 290 corresponding to 216 599 PDB entries and 731 924 different chains, representing an increase of 26% compared to the previous MobiDB version. PDB entries with only nucleic acid or ligand molecules are excluded. After combining PDB data at the protein level we collected confident ‘missing residues’ regions for ca. 22 839 proteins and ‘mobile’ regions for 2410 proteins using the MOBI algorithm (23).

### MobiDB-lite and prediction of ensemble properties

In MobiDB, predictions are generated with the MobiDB-lite software. In this release, we updated the MobiDB-lite software by integrating annotations of ‘*compact*’ and ‘*extended*’ ID regions. This classification is based on the value of the apparent Flory scaling exponent, $\nu$, predicted by the machine learning model developed by Tesei et al. (21). Specifically, we label ID regions with $\nu \le$0.475 as ‘compact’ and those with $\nu >$0.55 as ‘expanded’. These ranges correspond to the 5% most compact and the 32% most expanded of the human ID regions (21). Among compact human IDRs, we find examples such as the C-terminal domain of CTR9 (Q6PD62, $\nu$ = 0.412) and the N-terminal domain of FUS (P35637, $\nu$ = 0.467), whereas among the expanded ones, we find BASP1 (P80723, $\nu$ = 0.552) and prothymosin-α (P06454, $\nu$ = 0.592). The MobiDB-lite package has been completely rewritten to optimise its execution time which is now ten times faster. The calculation of the new labels does not significantly affect the execution time because it is fast and performed only for MobiDB-lite disordered regions with at least 30 residues.

### Function annotation

Function prediction is a challenging task, as highlighted by the recent results of the Critical Assessment of Automatic Function Annotation (CAFA) initiative (26). While functional annotations are typically assigned to entire proteins or their domains, disordered regions can possess independent functions. DisProt, for instance, assigns Gene Ontology (GO) (25) and Intrinsically Disordered Proteins Ontology (IDPO) terms to disordered regions based on experimental evidence. In MobiDB, we aim to capture and report functions explicitly associated with intrinsically disordered regions.

**Figure 1.**
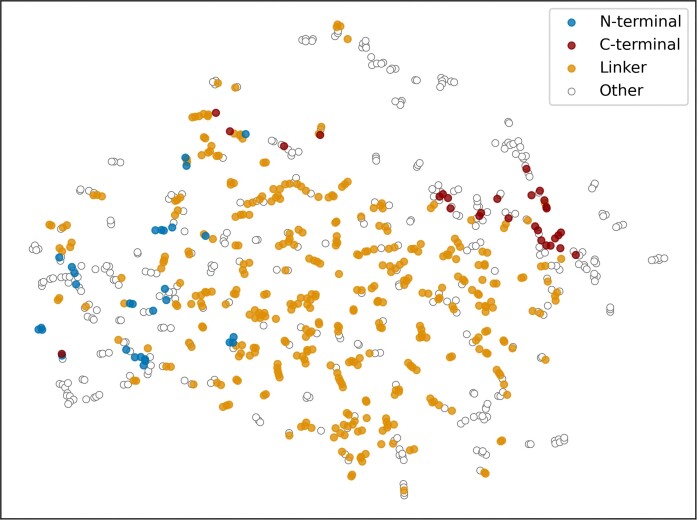
TSNE visualisation of IDRs with IDPO annotations in DisProt 2024_06 release (816 in total). The points are coloured based on the ‘entropic chain’ functions (IDPO:00501); i.e. ‘flexible linkers/spacers’ (IDPO:00502), ‘flexible N-terminal tail’ (IDPO:000503), ‘flexible C-terminal tail’ (IDPO:00504) or ‘Other’ if they were not annotated. The TSNE was done with the perplexity of 5, cosine distance metric, random state of 42 and 5000 iterations.

To achieve this, we employ three methods. i) A direct import of annotations from DisProt; this includes ‘*Molecular Function*’, ‘*Biological Process*’, and ‘*Cellular Component*’ annotations from GO, as well as ‘*Disorder Function*’ annotations from IDPO. ii) A homology-based annotation propagation; using sequence similarity inferred through BLAST (27), DisProt annotations are propagated to other proteins by comparison against the full sequences in UniProtKB. iii) An annotation transfer via protein embeddings; In this approach, we utilise the ProtT5-XL-UniRef50 protein language model (pLM) (28) to propagate functional annotations between similar IDRs. In order to obtain a representation for disordered regions, first we compute residue embeddings (vector representations from the last hidden layer of the pLM) of size 1024 for the entire protein. Then for each MobiDB-lite predicted ID region, we compute the average embedding of the residues within that region. Figure 1 presents a t-distributed stochastic neighbour embedding (TSNE) visualisation of all ID regions with IDPO annotations from the latest DisProt release (2024_06), highlighting three categories related to the entropic chain (IDPO:00501), flexible linkers/spacers (IDPO:00502), flexible N-terminal tails (IDPO:000503), and flexible C-terminal tails (IDPO:00504). We observe that the IDR embeddings related to the mutually exclusive classes ‘flexible N-terminal tail’ and ‘flexible C-terminal tail’ are separable, with the exception of few regions which corresponded to very short proteins. On the other hand, many linker regions can also be found in the tails of the sequence, despite not being annotated as terminal tails in DisProt. It is worth noting that DisProt contains a small amount of functionally annotated IDRs with respect to all the existing IDRs. Therefore Figure 1 does not resemble the actual distribution of all IDRs.

Following the methodology in (29), we employ a k-nearest neighbours (KNN) classification approach to transfer the GO Molecular Function and IDPO Disorder Function terms from the DisProt to the MobiDB-lite predicted ID regions. For each MobiDB-lite predicted ID region, the score for a functional annotation is calculated as an average of the neighbouring annotations, with the weights determined by the normalised distances. A distance threshold of 0.8 was chosen as a cut-off before the normalisation.

This approach was tested on the DisProt gold dataset, using the latest added proteins in the 2024_06 release as the test set and the 2023_12 release as reference. The distance metric employed was the cosine distance between vectors. The f-max was optimised with *K* = 10 and a score threshold of 0.44 for Molecular Function, and K = 5 with a score threshold of 0.59 for Disorder Function terms. F-max scores of 0.72 and 0.83 were achieved for Molecular Function and Disorder Function respectively, calculated at the IDR level following the CAFA challenge settings (30). To address the label imbalance issue—where most DisProt annotations involve binding (GO:0005488) and protein binding (GO:0005515)—a stricter score threshold of 0.9 was applied to filter these labels.

## Database content

**Figure 2.**
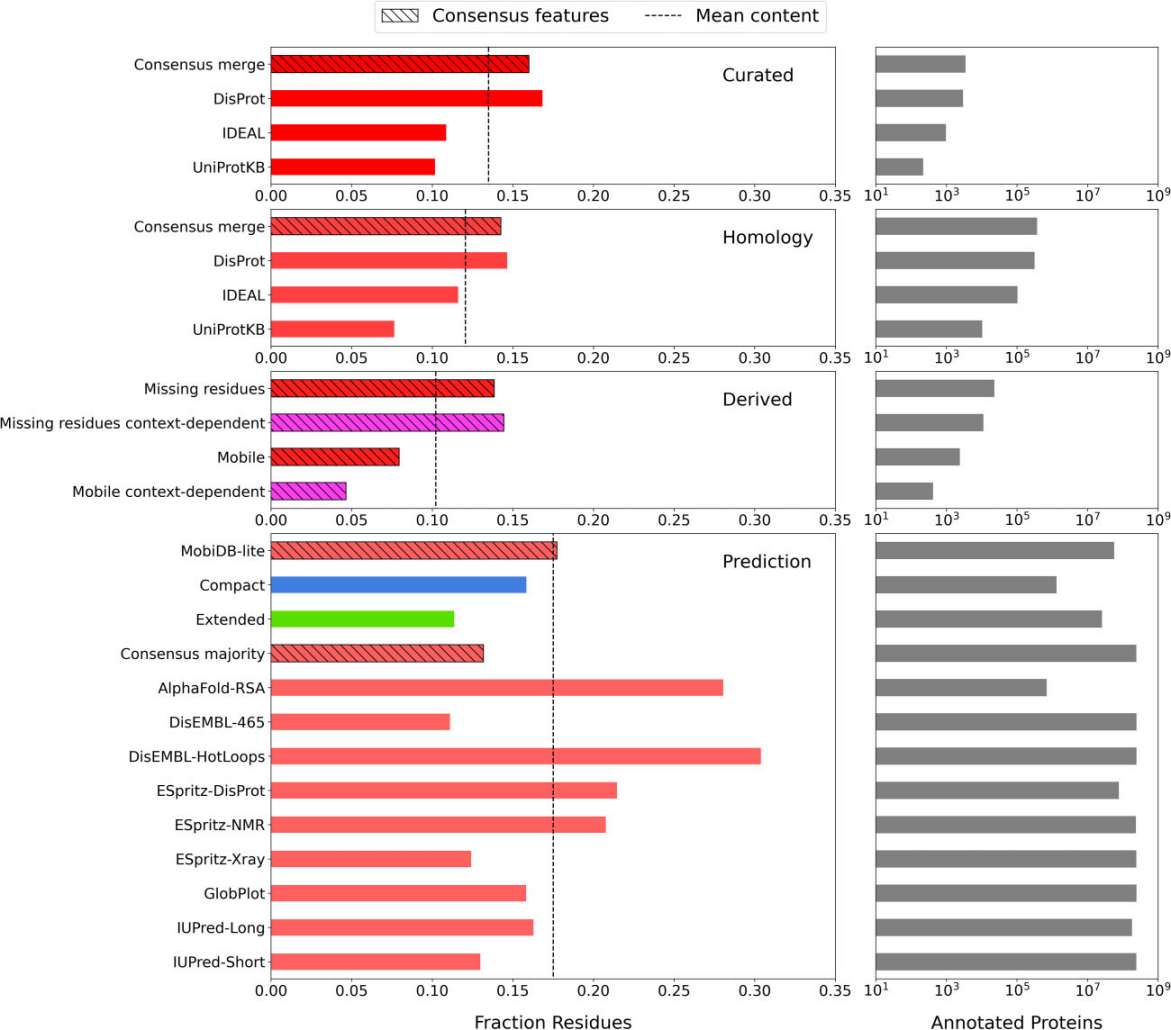
MobiDB statistics for disorder annotations by annotation evidence level. Different annotation levels (Curated, Homology, Derived, Prediction) are organised in different rows. Bar colours reflect those used in the MobiDB website. ‘*Extended*’ and ‘*compact*’ features corresponding to ensemble properties predictions integrated into MobiDB-lite are in blue and green. Context-dependent features, corresponding to PDB derived features in which the structural state depends on the experimental conditions, are coloured in magenta. Grey bars (plots in the second column) indicate the number of proteins with at least one annotated region. Note the scale of the x-axis is logarithmic. Prediction methods include MobiDB-lite (15), MobiDB-lite compactness subfeatures (‘Compact’, ‘Extended’) (21), AlphaFold-disorder (‘AlphaFold-RSA’) (31), DisEMBL (‘DisEMBL-465″, ‘DisEMBL-HotLoops’) (32), ESpritz (‘ESpritz-DisProt’, ‘ESpritz-NMR’, ‘ESpritz-Xray’) (33), GlobPlot (34) and IUPred (‘IUPred-Long’, ‘IUPred-Short’) (35). Derived annotations (‘Missing residues’, ‘Mobile’) are generated using the MOBI (23) and FLIPPER (24) software.

**Figure 3.**
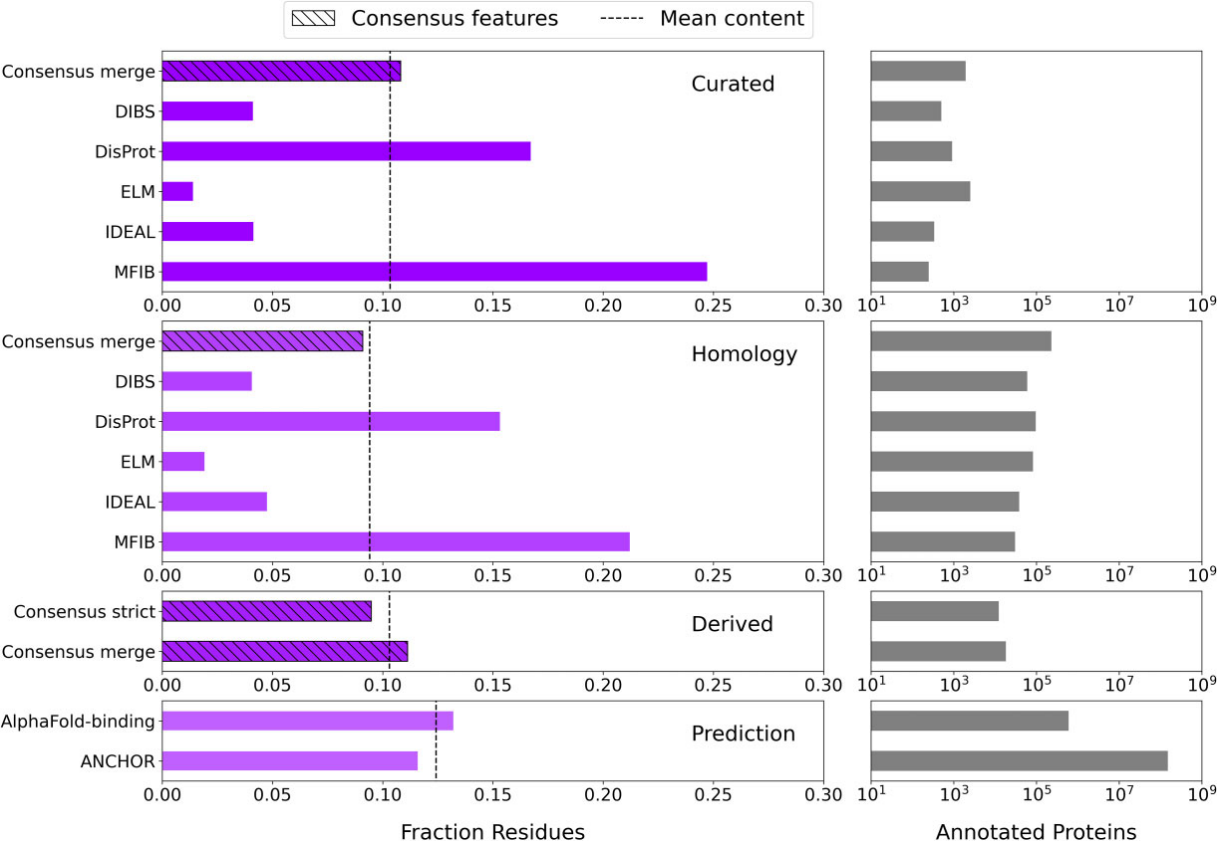
MobiDB statistics for linear interaction peptide (LIP) annotations by annotation evidence level. Same as Figure 2 but considering LIP annotations. Derived features are obtained from PDB data using the FLIPPER (‘Consensus strict’, ‘Consensus merge’) software (24). Prediction methods include AlphaFold-disorder (‘AlphaFold-binding’) (31) and ANCHOR (36).

In Figure 2, we reported the fraction of annotated residues and the number of annotated proteins for disorder features integrated in MobiDB, while in Figure 3 the same type of information is provided for Linear Interacting Peptide (LIP) annotations. Every row represents different confidence levels, namely curated annotations, derived from PDB data, propagated by homology and predictions. Some features are generated by combining other features to generate ‘*consensus*’ annotations (hatched bars in the figure). MobiDB offers different approaches to calculate consensus tracks, for example, merge indicates the union of the annotations at the protein level, whereas ‘*strict*’ and ‘*majority*’ indicate a consensus calculated on the ‘*vote*’ of the various integrated features applying a threshold of 90% and 50%, respectively. For curated disorder (Figure 2, ‘*Curated*’ row), the average content fraction (dashed vertical line) is coherent across different resources, whereas curated LIP annotations (Figure 3, ‘*Curated*’ row) diverge significantly indicating the integrated resources are capturing different aspects of binding, e.g. short linear motifs (SLiMs) by ELM (11). Significant differences are also observed for disorder predictions, MobiDB-lite, which is a consensus method integrating most of the other methods, notably aligns with the average of the series.

A large fraction of proteins (45%) are predicted to have well-solvated ID regions with ‘*extended*’ conformational ensembles ($\nu >$0.550), while only 2% are predicted to have ‘*compact*’ ID regions ($\nu \le$0.475). The number of proteins with AlphaFold disorder and LIP predictions is underestimated as we only process downloadable organisms from the AlphaFoldDB for a total of ca. 960000 proteins.

The fraction of residues is calculated over the total number of residues of annotated proteins, i.e. excluding those without any annotated region of that specific type. The dashed vertical lines indicate the mean value of the series. Hatched bars indicate consensus features.

The function annotations combining curated, propagated by homology and predicted terms, are provided for 47356025 different proteins. In Table 1 are shown the number of annotated proteins for the Molecular Function (MF) and Disorder Function (DF) namespaces of the Gene Ontology (GO) and Intrinsically Disordered Proteins Ontology (IDPO), respectively. While Cellular Components (CC) and Biological Process (BP) GO terms are also provided in MobiDB, they are not reported in Table 1, as the prediction pipeline is not executed for those namespaces as they represent functions that are too abstract and designed to be applied at the entire protein level and not at the region level. Notably, despite the most abundant annotations for the MF namespace being ‘binding’ functions, a large fraction of proteins are also predicted to perform ‘molecular adaptor activity’ (GO:0060090) and ‘molecular function regulator activity’ (GO:0098772).

**Table 1.** Functionally annotated proteins in MobiDB

Namespace	Term	Term name	Curated	Homology	Prediction
Disorder Function (IDPO)	IDPO:00502	Flexible linker/spacer	317	31714	23200290
	IDPO:00501	Entropic chain	24	1603	15123574
	IDPO:00504	Flexible C-terminal tail	30	2700	3967252
	IDPO:00503	Flexible N-terminal tail	26	2363	3634519
	IDPO:00025	Phosphorylation display site	122	5104	3191872
	IDPO:00024	Molecular recognition display site	13	1239	3023196
	IDPO:00505	Self-regulatory activity	4	542	2344651
	IDPO:00506	Self-inhibition	24	1620	1053900
	IDPO:00026	Acetylation display site	13	199	223003
	IDPO:00508	Self-assembly	24	1234	156345
Molecular Function (GO)	GO:0005515	Protein binding	642	45318	7259030
	GO:0003676	Nucleic acid binding	43	3862	3255313
	GO:0060090	Molecular adaptor activity	151	10959	2725151
	GO:0098772	Molecular function regulator activity	112	8314	2280943
	GO:0005488	Binding	0	0	1198483
	GO:0140693	Molecular condensate scaffold activity	41	2570	570237
	GO:0003723	RNA binding	40	1906	466434
	GO:0003677	DNA binding	41	2713	285069
	GO:0036094	Small molecule binding	35	3027	195155
	GO:0001069	Regulatory region RNA binding	6	489	182389

Only the top 10 terms of the Molecular Function and Disorder Function namespaces of the Gene Ontology (GO) and Intrinsically Disordered Proteins Ontology (IDPO) are reported. Counts are provided for each type of evidence, (i) ‘curated’, directly transferred from DisProt; (ii) ‘homology’, transferred by homology based on sequence similarity; and iii) ‘predicted’, annotated though our prediction pipeline.

## Website

MobiDB data is made available both through the MobiDB website and via a server API. Major achievements in the new MobiDB release include (i) an improvement of the API, which now leverages pagination and the RESTful paradigm to optimise bulk downloads; (ii) a restyled entry page, which better highlights disordered binding regions, the corresponding interaction partners and ID regions functions and (iii) improved documentation and training material about how to use the resource and information about the MobiDB project. We also included a controlled vocabulary section describing the identifiers of the different types of annotations. The vocabulary tab provides detailed explanations of how annotation identifiers are used, along with examples. Users can also download all possible triplets used in the database.

We have relocated the API page within the help page, which is now a tab called ‘*Programmatic access*.’ This page offers expanded documentation about the API and the database schema, as well as information about Bioschemas. We also introduced an OpenAPI Specification (formerly known as Swagger) page where the user can learn and test custom requests to the MobiDB API.

The about page has been updated to include information about the governance of MobiDB. This includes details about the MobiDB team, the role and contribution of the Scientific Advisory Board (SAB), and how the SAB operates. We have added a section that provides a link to a document containing Quality Assurance standard operating procedures (SOPs), the roadmap, the Resource Improvement Plan, and QA/QC implementation chapters. This document is a versioned Google Drive document and serves as a basis for the periodic SAB review. Additionally, the about page now features a funding tab that lists all the contributing projects, along with their corresponding start and end dates. Finally, we have created a ‘*License & Policy*’ page (previously ‘*License & Privacy*’) where we have documented our ELSI Policy and Equal Opportunity Research Support.

Finally, in order to provide a comprehensive description of the MobiDB resource, we created a section within the help page with links to training material including protocol articles (37), video tutorials, and more.

### Entry page

The entry page has been restyled to enhance the clarity of the database content and to highlight annotation features that might be overlooked because they are too hidden in the page.

Elements inside the page have been reorganised. Now the feature viewer is the first component to be rendered right after the entry header, therefore providing an immediate overview of the disorder (and binding) position in the protein sequence. Moreover, the first track of the feature viewer is always the disorder consensus in all tabs, therefore providing a means for comparing disorder with other types of annotations.

We created a new ‘*interaction*’ tab along with the ‘*overview*’, ‘*disorder*’ and ‘*binding*’ ones, which includes interactions information extracted from PDB complexes using the RING software. The same information was already available inside the binding tab but at the end of the page. Now ‘*binding*’ includes information about what are the disordered regions that can perform binding whereas ‘*interactions*’ includes information about the binders of the protein regardless if they bind a disordered region or not. The interaction tab allows the visualisation of PDB interactions directly in the structure viewer and lets the user colour the residues by clicking regions in the feature viewer.

The feature and sequence viewers have been completely reimplemented to improve rendering performance and customization. All molecular visualisation components used in MobiDB are designed for the Angular framework and are available at the URL: https://biocomputingup.github.io/ngx-mol-viewers/. The package also includes an Angular wrapper for the Mol* structure viewer (38).

### Browsing and searching data

MobiDB data can be retrieved using the browse page or the MobiDB API. The browse page mimics the API by reflecting the parameters in the page URL, therefore the same URL (except for the first part) can be used to request the same data from the server or copy-paste the URL in a new page. We included a new search field ‘*Feature exists*’ that lets the user retrieve entries with a specific feature. Due to limitations in the database management system, only a limited number of features are indexed and can be searched effectively. However, we indexed all consensus features so that when downloading a specific dataset, for example, all manually curated entries having the ‘*curated-disorder-merge*’ feature key, all subfeatures (‘*curated-disorder-disprot*’, ‘*curated-disorder-ideal*’, …) will be included by definition.

In order to simplify the understanding of the reliability of the underlying annotations we associated every annotation feature with a three-state flag (‘*gold*’, ‘*silver*’, no flag) and propagated that at the entry-level to facilitate the search, i.e. it is possible to retrieve all entries with at least one ‘gold’ annotation. In case a user needs disorder predictions for a sequence that is not available in UniProtKB and therefore in MobiDB, we provide a link in the home page to the CAID Prediction Portal (39) which can execute in parallel all methods evaluated in CAID (40).

### Server and API

The server backend has been refactored. The major limitation of the previous MobiDB version was the database timeout error triggered when downloading large amounts of data. We solved this problem by implementing database pagination, i.e. retrieving blocks of entries, the pages, iteratively. The search is effective even when the page is at the end of the searched list. This is possible thanks to the creation of database indexes that permit range searches. A new page can be retrieved in no time by providing a reference to the previous page. We delegate the client to iterate the pages and possibly concatenate the result in a single output. Pagination is transparent for the user when using the UI for browsing or downloading data, while it has to be embedded in a loop when using a custom client, i.e. a script. Information about the API endpoints are implemented and how to use them is provided in an OpenAPI Specification page (https://mobidb.org/help#swagger) and documented in ‘*protocol*’ articles (37) with specific examples and use cases.

## Conclusions and future work

Understanding ID proteins requires a shift from the sequence-structure-function relationships of folded proteins to the concept of ‘*sequence-ensemble-function*’ relationships. Exploring the complex conformational landscapes of ID regions offers insights into cellular biology, disease mechanisms, and the intricate interplay between protein sequences, structural ensembles and functional outcomes. Therefore, MobiDB is committed to integrating disorder information, including ensemble properties, to provide structural and functional insights.

As a result of a collaboration with the Kresten Lindorff-Larsen group, we integrated the prediction of compactness of disordered regions into MobiDB-lite and therefore MobiDB. Additionally, we worked on integrating region-based functional annotations as provided by DisProt and applying propagation algorithms that exploit sequence similarity and protein embeddings.

MobiDB is the core of the IDPcentral consortium and a number of European projects as well as global initiatives, including the ELIXIR IDP Community (41). In addition to online documentation, the MobiDB team organises online and in-person training courses in collaboration with other consortia, including ELIXIR and PhaseAge.

MobiDB, by standardising the way complementary annotations are combined, serves users who require curated disorder annotations, experimental data, and prediction results for known protein sequences as well as developers who want to derive reliable training datasets.

The aim of MobiDB in the near future is to improve the integration with primary data resources and the increase of metadata associated with every annotation, including experimental parameters as defined by the Minimum Information About a Disorder Experiment (MIADE) guidelines (42).

## Data Availability

All the data and link to used software are available at https://mobidb.org.
